# Imaging features and transcatheter treatment of a giant pulmonary arteriovenous malformation in an elderly patient

**DOI:** 10.1259/bjrcr.20150005

**Published:** 2015-10-08

**Authors:** Ashish Chawla, Suresh Balasubramanian Babu, Anbalagan Kannivelu, Sumer S Shikhare, Raymond Chung

**Affiliations:** Department of Diagnostic Radiology, Khoo Teck Puat Hospital, Yishun Central, Singapore

## Abstract

Pulmonary arteriovenous malformations (PAVMs) comprise an anomalous communication between the pulmonary arterial and systemic circulation. The drainage is usually into one of the pulmonary veins, although rare instances of direct drainage into the left atrium or inferior vena cava have been reported. The result is a high-flow, low-resistance, right-toleft shunt. Although considered uncommon, PAVMs are being diagnosed with increasing frequency in this era of enhanced cross-sectional imaging with CT for lung screening. There is a strong association between PAVMs and hereditary haemorrhagic telangiectasia (HHT); PAVMs are more commonly found in females, with a female to male ratio of 8:1. These have varying clinical presentation, with most symptomatic PAVMs being diagnosed in the first three decades of life. The most common mode of presentation is dyspnoea on exertion. Other reported symptoms are epistaxis, chest pain, cough and, in the event of rupture, haemoptysis. Endocarditis, stroke and brain abscess formation occur frequently in patients with undiagnosed HHT with PAVMs. A 76-year-old female, with a presumed clinical diagnosis of asthma, presented to the emergency department with worsening shortness of breath. The imaging studies revealed a giant PAVM and a radionuclide scan demonstrated a large right-to-left shunt, likely accounting for her symptoms. She underwent successful transcatheter embolization (TCE) with a vascular plug performed by the interventional radiology team. The aim of this case report is to describe the imaging findings and TCE treatment of a giant PAVM.

## Summary

Pulmonary arteriovenous malformations (PAVMs) comprise an anomalous communication between the pulmonary arterial and systemic circulation. The drainage is usually into one of the pulmonary veins, although rare instances of direct drainage into the left atrium or inferior vena cava have been reported. The result is a high-flow, low-resistance, right-to-left shunt. Although considered uncommon, PAVMs are being diagnosed more frequently given the increased use of cross-sectional imaging with CT for lung screening. There is a strong association between PAVMs and hereditary haemorrhagic telangiectasia (HHT); PAVMs are more commonly found in females, with a female to male ratio of 8:1. These have varying clinical presentation with most symptomatic PAVMs being diagnosed in the first three decades of life. The most common mode of presentation is dyspnoea on exertion. Other reported symptoms are epistaxis, chest pain, cough and, in the event of rupture, haemoptysis. Endocarditis, stroke and brain abscess formation occur frequently in patients with undiagnosed HHT with PAVMs. A 76-year-old female, with a presumed clinical diagnosis of asthma, presented to the emergency department (ED) with worsening shortness of breath. The imaging studies revealed a giant PAVM and a radionuclide scan demonstrated a large right-to-left shunt, likely accounting for her symptoms. She underwent successful transcatheter embolization (TCE) with a vascular plug performed by the interventional radiology team. The aim of this case report is to describe the imaging findings and TCE treatment of a giant PAVM.

## Clinical presentation

A 76-year-old female presented to the ED with a 2-week history of increasing breathlessness associated with cough. She denied any chest pain, fever or haemoptysis. She was presumptively diagnosed with asthma 5 years ago owing to recurrent episodes of breathlessness and was using a salbutamol inhaler without substantial symptomatic improvement. Her significant past history included diabetes, hypertension and gout. 6 months earlier, she had visited the ED for similar symptoms and was subsequently investigated for iron deficiency anaemia. An upper gastrointestinal endoscopy showed multiple arteriovenous malformations (AVMs) in the gastric fundus, antrum and second part of the duodenum. There was no family history of note. Her physical examination revealed bibasilar crackles and decreased air entry in the left lower hemithorax and mild pedal oedema. Oxygen saturation on room air was 93%. The 12-lead electrocardiogram and cardiac enzymes were within normal limits. Blood investigations reaffirmed iron deficiency anaemia with a haemoglobin of 8 g dl^−1^.

## Differential diagnoses

The main working clinical diagnosis was an acute exacerbation of asthma despite the absence of wheeze and poor response to nebulization. The clinical differential diagnoses included pneumonia and pulmonary embolism. Based on the chest radiograph showing an ill-defined solitary pulmonary opacity, the differential diagnosis was expanded to include malignancy, allergic bronchopulmonary mycosis (ABPM), sequestration and PAVM. Malignancy is always a concern in an elderly patient. ABPM is a well-recognized complication in patients with long-standing asthma. It can appear as an elongated opacity on the chest radiograph, similar to a bronchocoele. Pulmonary sequestration can also present as a solitary opacity in the lower lung. Although congenital in aetiology, it can remain undiagnosed until late adulthood. A demonstration of systemic vascular supply and drainage is diagnostic for sequestration. PAVMs are being increasingly diagnosed in adult patients and usually appear as an elongated, sharply demarcated opacity in the lower lungs. A demonstration of the vessels coursing from the opacity towards the hilum is considered diagnostic of PAVM.^1^ A CT scan helps to confirm the diagnosis, demonstrate multiplicity of the PAVMs if present as a spectrum of HHT, and provide the essential information for treatment planning.

## Imaging findings

**Figure 1.**
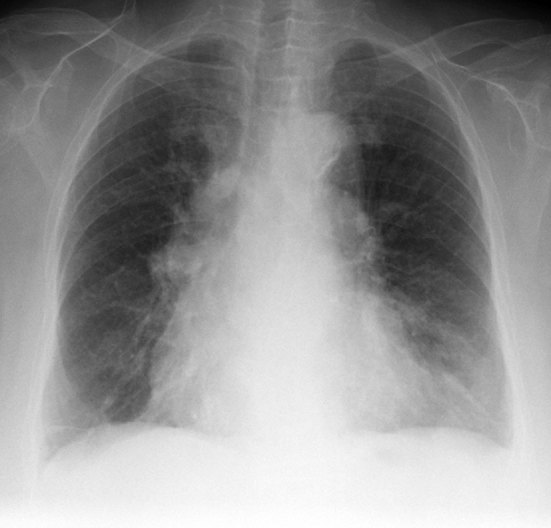
Chest radiograph shows a vague elongated opacity in the left lower lung.

**Figure 2.**
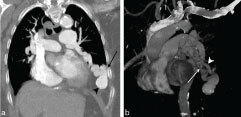
(a) Oblique coronal maximum intensity projection image shows a tortuous, enhancing arteriovenous malformation (black arrow) with a large aneurysm distally (arrowhead). (b) Three-dimensional CT reconstruction image demonstrates the feeding large tortuous pulmonary artery branch (white arrow) and a draining vein (arrowhead).

The patient’s chest radiograph showed an ill-defined, tortuous opacity in the left lower lung (Figure 1). A CT pulmonary angiogram, performed to rule out a pulmonary embolism, showed a large PAVM in the lingula of the left lobe with a giant aneurysmal component (Figure 2). The AVM was supplied by the lingular branch of the left pulmonary artery and drained into the left inferior pulmonary vein. The aneurysmal component of the AVM measured 2.5 cm and showed wall calcification. In those with a high clinical suspicion of a PAVM, some specialist centres recommend a non-contrast CT scan for both diagnosis and follow-up. This mitigates the risk of peripheral cannulation with subsequent paradoxical air embolism and its complications. The high intrinsic contrast afforded between the vasculature and the lung parenchyma on the non-contrast CT scan allows a safe diagnostic evaluation.

The scanner used was a 128-slice dual energy Siemens Somatom Definition Flash (Siemens Healthcare, Erlangen, Germany). The images were obtained in a caudocranial direction in suspended inspiration. The acquisition parameters were: 100 kVp for tube A and 140 kVp for tube B; combined applications to reduce exposure dose 4D (auto-dose modulation); 0.33 s rotation time; pitch of 0.9; 0.75 mm slice thickness with 128 × 0.6 mm beam collimation. The contrast medium used was Omnipaque 350 (GE Healthcare, Waukesha, WI) administered as a 60-ml bolus and 50-ml saline chaser at 4 ml s^−1^. Premonitoring was performed in the main pulmonary trunk with the trigger at 100 HU and the scan commenced 7 s after the trigger.

A two-dimensional echo revealed normal cardiac function. No other AVMs were identified on the screening CT aortogram. Functional information about the shunt fraction can be obtained either by radionuclide lung perfusion or contrast echo using agitated saline. However, false positives secondary to either a large patent foramen ovale or intracardiac shunts resulting in rare syndromes such as platypnoea–orthodeoxia may arise. Newer techniques, such as phase contrast MR angiogram to measure quantitative flow, without the need for contrast injection eliminate the risk of iatrogenic embolism. Radionuclide perfusion lung scan performed in our patient measured a right-to-left shunt fraction of 30.8%.

## Treatment and outcome

**Figure 3.**
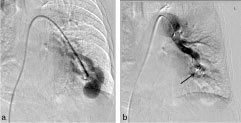
(a) Diagnostic selective lingular artery angiogram shows the arteriovenous malformation with an aneurysm. (b) After embolization with a vascular plug (black arrow), there is no further filling of the aneurysmal sac.

A direct catheter pulmonary angiography was performed in our patient via a transfemoral venous route, with strict attention paid to avoid an air embolus. The PAVM was successfully occluded with a 10 mm type 2 Amplatzer^®^ vascular plug) (Figure 3) placed distally in the solitary feeding artery. The patient’s oxygen saturation showed an immediate improvement and within 24 h, her breathlessness resolved. She was discharged from the hospital without any oxygen supplementation.

TCE has supplanted surgery as the treatment of choice for PAVMs given the reportedly high technical success yet low morbidity. All symptomatic PAVMs require treatment, although the guidelines for management of asymptomatic PAVMs remain unclear. The historical guideline of treating only those asymptomatic PAVMs that had a feeding artery diameter greater than 3 mm is no longer accepted.^2^ Studies have since shown that neurological complications, such as brain abscess formation from paradoxical embolization, are independent of the feeding diameter.^3,4^ The basic principle of TCE is to occlude the feeding artery close to the PAVM sac. Earlier experience of occluding the feeding artery has primarily been with long platinum coils. With the advancement in coil technology, alternatives such as detachable coils and microcoils have allowed even anatomically inhospitable PAVMs to be treated successfully. The more recent introduction of detachable occluders/vascular plugs, such as that used in this case, have allowed precise deployment and low recanalization rates with minimal morbidity.^5^^–^^7^

The radionuclide perfusion lung scan and the agitated saline contrast echo are useful screening tools to diagnose PAVMs in family members of a patient with HHT.^8,9^ As we considered the PAVM in this patient a part of an HHT spectrum, we have suggested screening of her family members. Furthermore, these studies provide a useful quantitative measure of treatment success following endovascular treatment. As such, we have recommended a follow-up perfusion lung scan at 6 months to assess the efficacy of treatment.

## Learning points

1. PAVMs may remain undiagnosed until late adulthood.
2. A non-contrast CT scan is sufficient to diagnose a PAVM.
3. During both peripheral venous and central pulmonary arterial cannulation, utmost care must be taken to avoid an air embolus, which could result in significant morbidity.
4. TCE is accepted as the standard method of treatment for PAVMs.
5. Radionuclide perfusion imaging helps in calculating the shunt fraction and is a useful objective tool to follow-up the efficacy of treatment.
6. Owing to the strong association of PAVMs with hereditary HHT, it is essential to screen the patients for similar lesions in the head, neck and abdomen by appropriate imaging modalities.
